# Effectiveness of Personalised Phone Calls and Short Message Service Reminders in Improving Patient Attendance at a Radiology Department

**DOI:** 10.7759/cureus.69568

**Published:** 2024-09-17

**Authors:** Majed Alturbag

**Affiliations:** 1 Department of Radiology, School of Nursing and Midwifery, Trinity College, University of Dublin, Dublin, IRL

**Keywords:** intervention, missed appointments, non-attendance, no-shows, radiology department

## Abstract

Background: Patients missing scheduled hospital appointments pose significant challenges, including resource waste and delayed patient care. This study evaluated the effectiveness of personalised reminder systems (phone calls and short message service (SMS)) in improving patient attendance rates at a radiology department.

Methods: The study was conducted at a hospital facility in Saudi Arabia. The intervention involved reminding 493 patients of their radiology appointments using their preferred method (phone call or SMS). Demographic, clinical, and other factors were considered in analysing the impact of these reminders on appointment attendance. Attendance rates before the intervention were further compared with attendance rates after the intervention to assess the effectiveness of the studied strategies.

Results: Patient reminders affected overall patient attendance, with a 5% improvement compared to the attendance rates before the intervention. Phone call reminders were found to be more effective than SMS, particularly among older patients (41-60 years). The attendance rate for patients receiving phone call reminders ranged from 35% to 85%, whereas those receiving SMS reminders had a 15-65% attendance range. The study indicated marital status and distance as key factors associated with attendance. Chi-square analysis also highlighted significant differences in attendance rates before and after the intervention, particularly among female patients, single and divorced individuals, and those with at least secondary education. Patients living more than 35 km from the hospital and those referred from other hospitals were more likely to miss appointments, irrespective of the intervention.

Conclusion: Personalised phone call reminders seem to be more effective than SMS in reducing missed appointments, especially among older patients. This study highlighted the importance of considering patient demographics and preferences in designing reminder systems to enhance healthcare appointment adherence.

## Introduction

Non-attendance at scheduled hospital appointments significantly impacts patient well-being, healthcare efficiency, and optimal utilisation of resources. Studies across various countries have indicated that 6.5-30% of appointments are unattended, leading to substantial financial and operational inefficiencies in clinical systems [1,2]. For instance, the UK’s National Health Service reported an estimated loss of £790 million annually due to non-attendance [3]. Beyond its financial impact, non-attendance exacerbates long waiting times, increases care delivery costs, underutilises staff and equipment, and adversely affects patient satisfaction and staff-patient relationships [2]. In addressing these challenges, various strategies have been explored. Some include offering incentives for attendance [4], imposing penalties for non-attendance, increasing appointment flexibility [5], providing transport solutions [6], and considering cultural factors in scheduling [7]. Apart from these strategies, sending appointment reminders, whether by post, telephone, or short message service (SMS), has emerged as a widely adopted and researched intervention [8-11]. The present intervention comprised the implementation of reminder systems, a subject that has garnered varying degrees of attention in existing literature. This study explored two primary reminder methods: SMS and telephone calls. Each method offers unique advantages and limitations. SMS reminders are automated and require minimal labour, providing a convenient and extensive reach. However, concerns about patient confidentiality and suitability for specific patient groups have been raised. Conversely, telephone call reminders offer a more personalised approach, potentially encouraging stronger connections between patients and hospital facilities.

The use of SMS reminders for appointment attendance in various health facilities offers several benefits over other reminder types. They can be automated, allowing large batches to be sent simultaneously to patients near and far. These reminders can be sent at any time, so patients can receive them at home or work [12,13]. However, concerns about the suitability of SMS have been raised. Moreover, patients cannot reschedule appointments directly through SMS and could ignore such messages. Previous studies, including both randomised controlled trials (RCTs) and observational studies, across various countries and clinical settings have indicated that SMS reminders generally reduce non-attendance rates, with attendance rate differences ranging from 3% to 43% [14-18].

Conversely, telephone call reminders provide a more personalised approach, creating stronger connections between patients and healthcare facilities. The International Telecommunication Union data confirm the widespread use of mobile phones, thereby rendering them a valuable tool in healthcare service delivery [1,19]. Personal phone call reminders, as per existing literature, improve patients’ intent to attend appointments, and further provide flexibility and a personal touch. Previous studies have highlighted the effectiveness of personal phone call reminders in reducing non-attendance rates, with differences in attendance rates ranging from 1.7% to 22% [11,20,21]. However, these calls incur higher costs and are typically restricted to standard working hours.

Existing studies have revealed that factors such as patient age, language spoken, and appointment day can affect the efficacy of reminders [16]. Moreover, the type of reminder used (personal or automated) has been found to impact patient attendance rates. As observed in studies like Parikh et al. (2010), personalised reminders made by clinic staff have a more substantial impact on attendance rates due to the emotional connection established with patients [22]. In contrast, automated reminders may be perceived as impersonal, as indicated by Satiani et al. (2009) [23], who found that automated telephone reminders led to higher non-attendance rates compared to no reminders. It seems that the effectiveness of reminders might be contingent on various factors, including patient characteristics and the nature of the intervention.

Another potential consideration is when reminders are sent. The timing of delivering phone call reminders, varying from one day to seven days before the appointment, has not been clearly correlated with non-attendance rates, suggesting that other factors may be more influential in patient attendance [18]. The frequency of reminders is another concern: while most studies employed a single reminder call, evidence is inconclusive on whether multiple reminders significantly affect non-attendance rates.

Despite these reasons behind the low attendance rate in hospitals, the intersection of technology and healthcare delivery is a focal point in modern medical research, particularly in optimising patient engagement and adherence to healthcare appointments. This research explored appointment adherence, examining the efficacy of reminder systems in reducing non-attendance rates in a radiology setting. Although the study assessed outcomes post-intervention, it was not a controlled trial but rather a prospective study without random assignment or a control group. A key component of the intervention is the personalisation of appointment scheduling and reminder systems, based on the premise that personalised appointment times and reminders can significantly reduce missed appointments. The primary research question for this study was whether personalised scheduling and reminders, including patient preference for the type of reminder (i.e., via SMS or phone call), reduce the number of missed appointments in radiology departments. The aims of the study were to develop and implement a system to reduce non-attendance rates, assess its effectiveness by tracking patient attendance, and compare attendance rates before and after the implementation of the intervention. In this study, attendance refers to patients who were present at their scheduled appointments, while non-attendance refers to patients who did not show up for their scheduled appointments. The study did not consider rescheduled appointments, as they were outside the scope of this research. This study contributes to the body of research on effective strategies for reducing missed appointments in clinical settings.

## Materials and methods

Participant access and ethical considerations

Prior to the intervention, ethical approval was obtained from both the Research Ethics Committee and the Regional Ethics Committee at the hospital. A critical aspect of this study was the role of the research assistant, who was exclusively responsible for accessing patient records and identities. The research assistant played a pivotal role in facilitating the intervention by providing necessary information to the researcher and sending appointment reminders to participants, ensuring privacy and confidentiality in the process.

Sample criteria and selection

The study employed two main inclusion criteria for participant selection. Participants had to be at least 18 years of age and were required to seek an in-person appointment at the department within the first two weeks of the recruitment period. The sample size for this study was calculated using the Raosoft online calculator (Raosoft, Inc., Seattle, WA), considering factors such as population size of 7,200 appointments, confidence level (95%), margin of error (2%), and response distribution (50%) [24]. Due to resource constraints, the final sample included 493 participants over 10 working days, increasing the margin of error to 4.26%, which remained within acceptable limits for the study. All patients seeking a radiology appointment during the specified period (October-December, 2022) were potential participants for this phase. Patients were approached by the research assistant about receiving an appointment reminder before their next scheduled appointment. The research assistant then asked those who agreed to confirm their contact details and inquired about their preference for receiving appointment reminders, i.e., via phone call or SMS. Data for those who agreed were obtained from the radiology department database and were included in the study. To raise awareness and encourage participation, posters with study details were displayed prominently in patient areas.

Intervention design

The intervention involved scheduling appointments as per the individual needs and preferences of patients, within the constraints of appointment availability. The intervention design also included a direct phone number on the appointment slip for the radiology department, allowing patients to reschedule if needed. Choices were offered to patients between two types of reminders, SMS or phone call, with reminders including necessary rescheduling contact details. The process for each patient involved confirming their contact details, preferred appointment times, and choice of reminder type. Reminders were sent 48 hours before the appointment, or 72 hours in advance for weekend appointments. Reminders were executed either via SMS or phone call and contained minimal patient information to maintain confidentiality. The content included the patient’s first name, the hospital name, and the appointment time and date (for example, “Dear Ali, this is a reminder about your appointment at the radiology department at 9:45 a.m. on 10 November”), with an additional request for appointment confirmation in SMS reminders.

Data collection

Data for this study were collected from the departmental database and case notes, encompassing appointments made from 11 October to 8 December, 2022. A structured data extraction instrument was developed to capture demographic details, clinical factors, reminder types, and attendance rates. This instrument was designed to collect data in a similar manner as it was collected before the implementation in the quantitative study. The form enabled the efficient recording of various data points, with age being the only variable requiring numerical input. The research assistant documented a total of 493 appointments using a structured data extraction form. Each patient’s data were coded for anonymity, with numerical values assigned to various variable attributes. This approach not only facilitated data organisation but also allowed for the tracking of any missing values. To ensure the utmost privacy and confidentiality, a rigorous protocol was established. Each patient was assigned a unique key code, and all identifiable data were removed before the forms were handed to the researcher. Post-data analysis, these unique codes were destroyed to maintain confidentiality. The extracted data were then entered into a Microsoft Excel spreadsheet (Microsoft Corporation, Redmond, WA) by the researcher, following a dichotomous approach.

Data analysis

Dataset accuracy was first verified by cross-checking the number of documents against the extraction forms obtained. Each variable was then assigned a numerical value and entered into Microsoft Excel. This step was crucial to ensure no missing data were present before importing the data into SPSS version 23 (IBM Corp., Armonk, NY) for further analysis. The intervention data, encompassing both phone calls and SMS reminders, were then analysed using two primary tools: SPSS for descriptive analysis and the R programming language (version 4.1.0; R Foundation for Statistical Computing, Vienna, Austria) in the RStudio (Posit, Boston, MA) environment for inferential statistical analysis [25]. This dual approach facilitated a comprehensive analysis, ranging from descriptive statistics to bivariate analysis, examining the relationship between various independent variables (demographic, clinical, and other factors) and the dependent variable (non-attendance).

The initial analysis used descriptive statistics to quantitatively describe each variable. This analysis aimed to identify the frequency and percentage of each variable, specifically focusing on attendance rates in different reminder groups. These frequencies were then compared across the two types of intervention reminders to understand the patterns of missed appointments across different categories.

Bivariate analysis was employed to examine the relationships between various independent and dependent variables, assessing how each variable influenced patient outcomes. Independent variables were categorised into demographic characteristics (age group, gender, nationality, marital status, education), clinical factors (request sources, radiology tests), and other factors (distance, intervention type, service payment). A chi-square (χ2) test of independence, a non-parametric test, was conducted using SPSS to determine the association between these variables and patient attendance. Additionally, a two-proportion Z-test was conducted to compare non-attendance rates between the pre- and post-intervention phases, ensuring statistical comparability of the populations and determining the intervention's impact on attendance. Further, to compare attendance rates before and after the intervention based on various factors, the chi-square test was conducted in R, using the chisq.test function for categorical variables. The statistical significance of attendance and non-attendance rates for each variable category was analysed at α = 0.05 level of significance. R tool was pivotal in performing a 2 x 2 chi-square analysis for the pre- and post-intervention results, and its aim was to reduce the complexity of the data.

This study focussed specifically on analysing patient attendance rates following the implementation of reminder strategies (SMS and phone calls) at a radiology department. However, the data used for comparative purposes in this study have a foundational basis in a quantitative study, which is scheduled for separate publication. This quantitative study involved a detailed analysis of patient attendance rates before the introduction of the reminder strategies. In the present intervention analysis, key metrics and findings from the quantitative study were utilised as a baseline for comparison. The metrics, including attendance rates and relevant demographics, were critical in providing context for and depth to the findings. The statistical methods used for analysis in both studies are consistent, ensuring comparability and reliability of the results.

## Results

This study involved developing, implementing, and evaluating a system that allowed patients to schedule appointments at their convenience and receive timely reminders. The effectiveness of the intervention was assessed by considering various demographic, clinical, and other factors. A total of 493 participants were enrolled to evaluate the impact of personalised scheduling and reminders on patient attendance rates.

The analysis categorised participants into various demographic groups. The age distribution indicated that the largest group, comprising 183 (37.1%) patients, was between 21 and 40 years old. The gender distribution was fairly balanced, with males constituting 250 (50.7%) and females constituting 243 (49.3%) of the sample (Table 1). Many participants were married (276, 56%) and held a degree-level education (52.1%, 257/493), with most being of Saudi nationality (318, 64.5%). Clinical profiles suggested that ultrasound tests were the most common in this sample. Notably, 392 (79.5%) of patients did not need to pay for services, likely due to insurance coverage. Of all patients, 289 (58.6%) chose phone call reminders, while 204 (41.4%) preferred SMS reminders. The analysis revealed that 406 (82.4%) of patients attended their scheduled appointments, whereas 87 (17.6%) (87/493) missed their appointments.

**Table 1:** Participants' profile and descriptive analysis after the implementation of intervention (N = 493).

Variables	Categories	Frequency (%)
Age group, mean ± SD (46.64 ± 17.621)	20 years or less	21 (4.3)
	21 to 40 years	183 (37.1)
	41 to 60 years	175 (35.5)
	61 to 80 years	99 (20.1)
	Above 80 years	15 (3.0)
Gender	Male	250 (50.7)
	Female	243 (49.3)
Marital status	Single	77 (15.6)
	Married	276 (56.0)
	Divorced	100 (20.3)
	Widowed	40 (8.1)
Education	Primary education	31 (6.3)
	Secondary education or diploma	140 (28.4)
	Degree	257 (52.1)
	Higher degree	65 (13.2)
Nationality	Saudi	318 (64.5)
	Non-Saudi	175 (35.5)
Request source	In-patient	55 (11.2)
	Out-patient	212 (43.0)
	Referred from another hospital	226 (45.8)
Distance	Less than 35 km away from the hospital (Buraydah)	330 (66.9)
	Greater than or equal to 35 km away from the hospital	163 (33.1)
Radiology test	Ultrasound	128 (26.0)
	MRI	44 (8.9)
	CT scan	77 (15.6)
	Angiogram	69 (14.0)
	Nuclear medicine	68 (13.8)
	Special investigation	43 (8.7)
	Bone density scan	31 (6.3)
	Mammogram	33 (6.7)
	No payment for the service	392 (79.5)
	Payment for the service	101 (20.5)
	Phone call	289 (58.6)
	Short message service (SMS)	204 (41.4)
	Did not attend	87 (17.6)
	Attended	406 (82.4)

**Figure 1:**
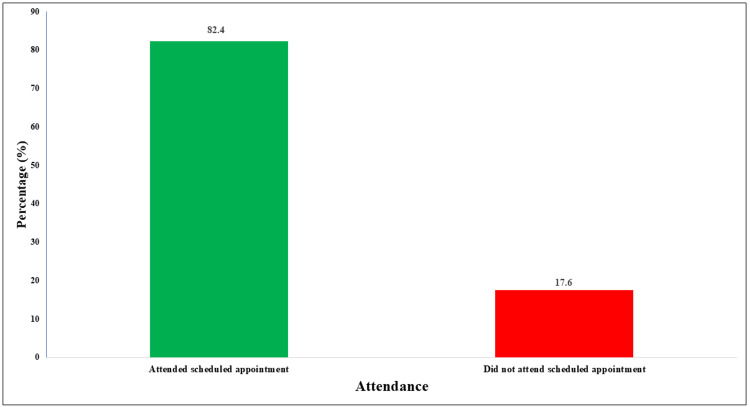
Percentage of attendance and non-attendance at scheduled appointments after the intervention.

An analysis of the data after the intervention revealed notable findings regarding appointment adherence in the radiology department. A significant majority (406, 82.4%) of patients attended their appointments, while 87 (17.6%) missed their appointments (Figure 1). This attendance rate represents a 5% improvement compared to the attendance rates before the implementation of the intervention, where the attendance rate was 77.4%, indicating a successful reduction in missed appointments.

The study further investigated the effectiveness of the intervention based on the type of reminder preferred by patients. A descriptive analysis classified the number of participants according to their chosen reminder method. Chi-square tests were then conducted to explore the relationship between the reminder preferences and various demographic, clinical, and other factors. Table 2 includes a detailed breakdown of these associations. Notably, age emerged as a significant factor. Patients aged 41 to 60 years were more likely to respond positively to phone call reminders, with 107 (71.8%) in this age group attending their appointments when reminded via phone call. In contrast, younger patients aged 21 to 40 years had a higher attendance rate of 96 (64.4%) when reminded through SMS. Out of SMS and phone calls, gender-wise analysis indicated that 58% of male and 59.3% of female patients were reminded via phone call. Among these, 84.7% of females and 83% of males attended when reminded by phone call. Interestingly, more male patients preferred SMS reminders than did females. Marital status also influenced reminder preferences. A majority of (168, 60.9%) married patients opted for phone call reminders, with a notable attendance rate of 91.6%. Conversely, among married patients reminded via SMS, 91 out of 108 (84.25%) attended their appointments. This suggested a higher effectiveness of phone call reminders in this demographic. Considering education, 126/149 (86.92%) of patients with degree-level education attended their appointments when reminded. Patients with secondary education or a diploma exhibited a preference for phone calls (56%), with an 83.5% attendance rate, compared to a 74% attendance rate for those receiving SMS reminders. Nationality and referral source were other factors influencing reminder preference. Saudi patients predominantly preferred phone calls, with 155/193 (80.3%) attending their appointments. Out-patients, on the other hand, were more responsive to SMS reminders, with a 77/95 (81.05%) attendance rate. Proximity to the hospital also played a role, as patients living close to the hospital had a 179/195 (91.7%) likelihood of attending appointments when reminded via phone call whereas 117/135 (86.6%) when reminded by SMS.

**Table 2:** Bivariate analysis of patients based on the intervention type. **: P-value is significant at <0.01.

Variables	Groups		Intervention type		Chi-square	p-value
			Phone call (n = 289)	SMS (n = 204)		
			n (%)			
Age group	20 or less	Total	7 (33.3)	14 (66.7)	82.359	<0.001**
		Attended	7 (36.8)	12 (63.2)		
		Did not attend	0	2 (100)		
	21 to 40	Total	65 (35.5)	118 (64.5)		
		Attended	53 (35.6)	96 (64.4)		
		Did not attend	12 (35.3)	22 (64.7)		
	41 to 60	Total	123 (70.3)	52 (29.7)		
		Attended	107 (71.8)	42 (28.2)		
		Did not attend	16 (61.5)	10 (38.5)		
	61 to 80	Total	82 (82.8)	17 (17.2)		
		Attended	67 (85.9)	11 (14.1)		
		Did not attend	15 (71.4)	6 (28.6)		
	81 and above	Total	12 (80)	3 (20)		
		Attended	9 (81.8)	2 (18.2)		
		Did not attend	3 (75)	1 (25)		
Gender	Male	Total	145 (58)	105 (42)	0.81	0.777
		Attended	121 (58.7)	85 (41.3)		
		Did not attend	24 (54.5)	20 (45.5)		
	Female	Total	144 (59.3)	99 (40.7)		
		Attended	122 (61)	78 (39)		
		Did not attend	22 (51.2)	21 (48.8)		
Marital status	Single	Total	34 (44.2)	4355.8)	8.596	0.35
		Attended	26 (44.8)	32 (55.2)		
		Did not attend	8 (42.1)	11 (57.9)		
	Married	Total	168 (60.9)	108 (39.1)		
		Attended	154 (62.9)	91 (37.1)		
		Did not attend	14 (45.2)	17 (54.8)		
	Divorced	Total	60 (60)	40 (40)		
		Attended	43 (58.9)	30 (41.1)		
		Did not attend	17 (63)	10 (37)		
	Widowed	Total	27 (67.5)	13 (32.5)		
		Attended	20 (66.7)	10 (33.3)		
		Did not attend	7 (70)	3 (30)		
Education	Primary education	Total	22 (71)	9 (29)	3.788	0.285
		Attended	15 (71.4)	6 (28.6)		
		Did not attend	7 (70)	3 (30)		
	Secondary education or diploma	Total	87 (62.1)	53 (37.9)		
		Attended	72 (64.9)	39 (35.1)		
		Did not attend	15 (51.7)	14 (48.3)		
	Degree	Total	145 (56.4)	112 (43.6)		
		Attended	126 (58.1)	91 (41.9)		
		Did not attend	19 (47.5)	21 (52.5)		
	Higher degree	Total	35 (53.8)	30 (46.2)		
		Attended	30 (52.6)	27 (47.4		
		Did not attend	5 (62.5)	3 (37.5)		
Nationality	Saudi	Total	193 (60.7)	125 (39.3)	0.015	0.903
		Attended	155 (60.1)	103 (39.9)		
		Did not attend	38 (63.3)	22 (36.7)		
	Non-Saudi	Total	96 (54.9)	79 (45.1)		
		Attended	88 (59.5)	60 (40.5)		
		Did not attend	8 (29.6)	19 (70.4)		
Request source	In-patient	Total	36 (65.5)	19 (34.5)	1.622	0.445
		Attended	32 (66.7)	16 (33.3)		
		Did not attend	4 (57.1)	3 (42.9)		
	Out-patient	Total	117 (45.5)	95 (54.5)		
		Attended	102 (57)	77 (43)		
		Did not attend	15 (45.5)	18 (54.5)		
	Referred from other hospital	Total	136 (60.2)	90 (39.8)		
		Attended	109 (60.9)	70 (39.1)		
		Did not attend	27 (57.4)	20 (42.6)		
Distance	Buraydah	Total	195 (59.1)	135 (40.9)	0.175	0.676
		Attended	179 (60.5)	117 (39.5)		
		Did not attend	16 (47.1)	18 (52.9)		
	Greater than or equal to 35 km away from the hospital	Total	94 (57.7)	69 (42.3)		
		Attended	64 (58.2)	46 (41.8)		
		Did not attend	30 (56.6)	23 (43.4)		
Radiology test	Ultrasound	Total	77 (60.2)	51 (39.8)	4.584	0.711
		Attended	64 (58.7)	45 (41.3)		
		Did not attend	13 (68.4)	6 (31.6)		
	MRI	Total	27 (61.4)	17 (38.6)		
		Attended	24 (63.2)	14 (36.8)		
		Did not attend	3 (50)	3 (50)		
	CT scan	Total	35 (45.5)	42 (54.5)		
		Attended	31 (50.8)	30 (49.2)		
		Did not attend	4 (25)	12 (75)		
	Angiogram	Total	46 (66.7)	23 (33.3)		
		Attended	39 (66.1)	20 (33.9)		
		Did not attend	7 (70)	3 (30)		
	Nuclear medicine	Total	40 (58.8)	28 (41.2)		
		Attended	31 (60.8)	20 (33.9)		
		Did not attend	9 (52.9)	8 (47.1)		
	Special investigation	Total	23 (53.5)	20 (46.5)		
		Attended	19 (54.3)	16 (45.7)		
		Did not attend	4 ( (50)	4 (50)		
	Bone density scan	Total	20 (64.5)	11 (35.5)		
		Attended	17 (65.4)	9 (34.6)		
		Did not attend	3 (60)	2 (40)		
	Mammogram	Total	21 (63.6)	12 (36.4)		
		Attended	18 (66.7)	9 (33.3)		
		Did not attend	3 (50)	3 (50)		
Payment mode	No payment	Total	226 (57.7)	166 (42.3)	2.728	0.099
		Attended	188 (57.8)	137 (42.2)		
		Did not attend	38 (56.7)	29 (43.3)		
	Payment	Total	63 (62.4)	38 (37.6)		
		Attended	55 (67.9)	26 (32.1)		
		Did not attend	8 (40)	12 (60)		

The mode of payment for services was another influential factor, with 226 (57.7%) of patients who were not required to pay for services preferring phone calls and exhibiting an attendance rate of 188 (83.1%). Regarding clinical factors, ultrasound was the most common test among appointments. For ultrasound appointments, 64 (58.7%) preferred phone call reminders, while 45 (41.3%) chose SMS. Non-attendance rate was higher for ultrasound and CT scans among all other radiology tests. Generally, phone call reminders proved more effective in reducing non-attendance, though this might be influenced by various demographic, clinical, or other factors.

Bivariate analysis

The purpose of this analysis was to ascertain whether the introduction of intervention reminders influenced patient non-attendance rates. In this study, a notable shift was observed in the association of demographic characteristics with patient attendance. The analysis revealed that only marital status had a significant association with attendance among demographic characteristics (p < 0.001). Variables such as gender, nationality, education, and age group did not demonstrate a statistically significant relationship with patient attendance after the intervention (Table 3).

**Table 3:** Bivariate analysis using chi-squared test between variables belonging to demographic characteristics and attendance (n = 493). **: P-value is significant at <0.01.

Variables	Groups	Attendance		Total number per group	Chi-square	p-value
		Attended (n = 406)	Did not attend (n = 87)			
		n (%)				
Gender	Male	206 (50.7)	44 (50.6)	250	.001	0.536
	Female	200 (49.3)	43 (49.4)	243		
Nationality	Saudi	258 (63.5)	60 (69.0)	318	13.616	0.092
	Non-Saudi	148 (36.5)	27 (31.0)	175		
Marital status	Single	58 (14.3)	19 (21.8)	77	17.940	<0.001**
	Married	245 (60.3)	31 (35.6)	276		
	Divorced	73 (18.0)	27 (31.0)	100		
	Widowed	30 (7.4)	10 (11.5)	40		
Education	Primary education	21 (5.2)	10 (11.5)	31	7.502	0.57
	Second education or diploma	111 (27.3)	29 (33.3)	140		
	Degree	217 (53.4)	40 (46.0)	257		
	Higher degree	57 (14.0)	8 (9.2)	65		
Age group	20 or less	19 (4.7)	2 (2.3)	21	3.706	0.447
	21 to 40	149 (36.7)	34 (39.1)	183		
	41 to 60	149 (36.7)	26 (29.9)	175		
	61 to 80	78 (19.2)	21 (24.1)	99		
	Above 80	11 (2.7)	4 (4.6)	15		

Analysis of other factors

Table 4 presents the analysis of other factors, such as distance from the hospital, that might have influenced patient attendance. Distance emerged as a significant factor; patients residing within 35 km of the hospital were more likely to attend their appointments after the intervention. However, variables like service payment and type of intervention (phone call or SMS) did not exhibit a significant association with attendance status (Table 5).

**Table 4:** Association between other factors and attendance (n = 493). **: P-value is significant at <0.01.

Variables	Groups	Attendance		Total number per group	Chi-square	p-value
		Attended (n = 406)	Did not attend (n = 87)			
		n (%)				
Distance	Less than 35 km away from the hospital (Buraydah)	296 (72.9)	34 (39.1)	330	37.042	<0.001**
	Greater than or equal to 35 km away from another hospital	110 (27.1)	53 (60.9)	163		
Service payment	No payment for service	325 (80.0)	67 (77.0)	392	0.406	0.524
	Pay for the service	81 (20.0)	20 (23.0)	101		

**Table 5:** Association of intervention type and attendance (n = 493).

Variables	Groups	Attendance		Total number per group	Chi-square	p-value
		Attended (n = 406)	Did not attend (n = 87)			
Intervention type	Phone call	243 (59.9)	46 (52.9)	289	1.438	0.140
	SMS	163 (40.1)	41 (47.1)	204		

Analysis of clinical factors

The study analysis also extended to clinical factors, including the source of the appointment request and the type of radiology test. As detailed in Table 6, none of these clinical factors were found to be significantly associated with attendance status. This finding suggests that clinical aspects, such as the nature of the radiology test or the referral source, did not significantly influence the likelihood of a patient attending their scheduled appointment following the intervention.

**Table 6:** Association between clinical factor and attendance (N = 493).

Variables	Groups	Attendance		Total number per group	Chi-square	p-value
		Attended (n = 406)	Did not attend (n = 87)			
		n (%)				
Request source	In-patient in King Fahad Hospital	48 (11.8)	7 (8.0)	55	3.090	0.213
	Out-patient in King Fahad Hospital	179 (44.1)	33 (37.9)	212		
	Referred from another hospital	179 (44.1)	47 (54.0)	226		
Radiology test	Ultrasound	109 (26.8)	19 (21.8)	128	4.784	0.686
	MRI	38 (9.4)	6 (6.9)	44		
	CT scan	61 (15.0)	16 (18.4)	77		
	Angiogram	59 (14.5)	10 (11.5)	69		
	Nuclear medicine	51 (12.6)	17 (19.5)	68		
	Special investigation	35 (8.6)	8 (9.2)	43		
	Bone density scan	26 (6.4)	5 (5.7)	31		
	Mammogram	27 (6.7)	6 (6.9)	33		

Comparative analysis of before and after intervention phases

Before assessing the effectiveness of the intervention by comparing non-attendance rates before and after intervention, it was critical to ensure that the populations from both phases were statistically comparable. This would allow any observed changes in non-attendance to be attributed to the intervention rather than differences in the populations themselves. A two-proportion Z-test was employed to compare non-attendance rates. The non-attendance rate before intervention was 22.59%, while after intervention, it dropped to 17.65%. The test resulted in a Z-statistic of 2.387, which exceeded the critical value of 1.96 at a 95% confidence level, indicating that the difference in non-attendance rates was statistically significant. This finding justifies proceeding with further comparative analysis of baseline characteristics, ensuring that any observed changes in attendance were the result of the intervention rather than differences between the populations at the outset.

Utilising the chi-square test of independence, the study analysed the influence of the intervention on attendance patterns in the context of various categorical variables. Table 7 presents the gender-specific findings. Female patients demonstrated a significant change in attendance from pre- to post-intervention, unlike their male counterparts. This result suggests that the intervention had a more noticeable impact on female than male attendance rates. Attendance rates among Saudi nationals were significantly impacted by the intervention, indicating a positive response to the reminder system. Considering marital status, no significant differences were found in the attendance rates of married and widowed patients before and after the intervention. In contrast, single and divorced patients demonstrated a notable shift in their attendance rates before and after intervention (p < 0.05), suggesting a significant impact of the intervention on these groups. The chi-squared analysis indicated that attendance rates among patients with at least secondary education or a degree were closely linked with intervention effectiveness with p = 0.007285 and p = 0.002353, respectively. Regarding age, the group aged 20 years or less had a highly significant change in attendance rates. Similarly, the 21-40 years age group exhibited a significant change in pre- and post-intervention attendance. A notable finding was the significant relationship in attendance rates for patients living more than 35 km away from the hospital. Despite the intervention, these patients were less likely to attend, mirroring the pattern observed before the intervention. Conversely, patients not required to pay for services had a shift in attendance rates, indicating a greater likelihood of attendance post intervention. The analysis also considered the source of referral, including in-patients, out-patients, and patients referred from other hospitals. Attendance rates for patients referred from other hospitals significantly shifted after the intervention. Among various radiology tests, the rise in MRI test attendance rates was significantly resulted due to the intervention.

**Table 7:** Attendance rates comparative analysis before and after the intervention. *: P-value is significant at <0.05. **: P-value is significant at <0.01.

Variables	Groups	Pre-intervention		Post-intervention		Chi-square	p-value
		Attended	Did not attend	Attended	Did not attend		
		(n = 1535)	(n = 448)	(n = 406)	(n = 87)		
		n (%)		n (%)			
Gender	Male	788 (51.3)	185 (41.29)	206 (50.7)	44 (50.6)	0.26	0.069
	Female	747 (48.6)	263 (58.7)	200 (49.3)	43 (49.4)	7.38	0.006**
Nationality	Saudi	951 (61.9)	312 (69.6)	258 (63.5)	60 (69.0)	4.807	0.028
	Non-Saudi	584 (38.0)	136 (30.3)	148 (36.5)	27 (31.0)	1.1317	0.287
Marital status	Single	286 (18.6)	166 (37.0)	58 (14.3)	19 (21.8)	4.201	0.0404*
	Married	946 (61.6)	123 (27.4)	245 (60.3)	31 (35.6)	0.0163	0.898
	Divorced	184 (11.9)	114 (25.44)	73 (18.0)	27 (31.0)	4.146	0.04173*
	Widowed	119 (7.7)	45 (10.0)	30 (7.4)	10 (11.5)	0.097	0.7553
Education	Primary education	117 (7.6)	104 (23.2)	21 (5.2)	10 (11.5)	2.404	0.121
	Second education or diploma	548 (35.7)	258 (57.5)	111 (27.3)	29 (33.3)	7.2013	0.007285**
	Degree	637 (41.4)	61 (13.6)	217 (53.4)	40 (46.0)	9.2515	0.002353**
	Higher degree	233 (15.1)	25 (5.5)	57 (14.0)	8 (9.2)	0.38787	0.5334
Age group	20 or less	59 (3.8)	46 (10.2)	19 (4.7)	2 (2.3)	8.7231	0.0031**
	21 to 40	438 (28.5)	160 (35.7)	149 (36.7)	34 (39.1)	5.0178	0.02509*
	41 to 60	537 (34.9)	128 (28.5)	149 (36.7)	26 (29.9)	1.7841	0.1816
	61 to 80	393 (25.6)	92 (20.5)	78 (19.2)	21 (24.1)	0.26507	0.6067
	Above 80	108 (7.0)	22 (4.9)	11 (2.7)	4 (4.6)	0.08676	0.3516
Distance	Less than 35 km away from the hospital (Buraydah)	1041 (67.8)	94 (20.9)	296 (72.9)	34 (39.1)	1.3097	0.2524
	Greater than or equal to 35 km away from another hospital	494 (32.1)	354 (79.0)	110 (27.1)	53 (60.9)	4.8429	0.02776*
Service payment	No payment for service	981 (63.9)	314 (70.0)	325 (80.0)	67 (77.0)	8.8116	0.002993**
	Pay for the service	554 (36.0)	134 (29.9)	81 (20.0)	20 (23.0)	0.005931	0.9386
Request source	In-patient in King Fahad Hospital	209 (13.6)	29 (0.06)	48 (11.8)	7 (8.0)	0.012196	0.9121
	Out-patient in King Fahad Hospital	700 (45.6)	123 (27.4)	179 (44.1)	33 (37.9)	0.0507	0.8218
	Referred from another hospital	626 (40.7)	296 (66.0)	179 (44.1)	47 (54.0)	11.077	0.0008739**
Radiology test	Ultrasound	424 (827.6)	78 (17.4)	109 (26.8)	19 (21.8)	0.0377	0.846
	MRI	234 (15.2)	119 (26.5)	38 (9.4)	6 (6.9)	7.3086	0.006862**
	CT scan	228 (14.8)	82 (18.3)	61 (15.0)	16 (18.4)	1.0495	0.3056
	Angiogram	90 (5.8)	13 (2.9)	59 (14.5)	10 (11.5)	0.12492	0.7238
	Nuclear medicine	161 (10.4)	39 (8.7)	51 (12.6)	17 (19.5)	0.9287	0.3352
	Special investigation	180 (11.7)	48 (10.7)	35 (8.6)	8 (9.2)	0.13224	0.7161
	Bone density scan	125 (8.1)	31 (6.9)	26 (6.4)	5 (5.7)	0.23304	0.6293
	Mammogram	93 (6.0)	38 (8.4)	27 (6.7)	6 (6.9)	1.5737	0.2097

## Discussion

The integration of intervention reminders in radiology settings, and particularly its effect on appointment adherence, has been a subject of considerable interest and research. This study explored the efficacy of such reminders, specifically SMS and phone call reminders, in reducing missed appointments in the radiology department of the hospital. The importance of addressing missed appointments lies not only in reducing healthcare costs but also in enhancing patient outcomes. The intervention in this study allowed patients to select a suitable date and time for their appointment and provided them with a reminder close to the appointment date. The implementation of these reminders resulted in a 5% increase in appointment attendance, from 77.4% to 82.4%. This finding highlights the effectiveness of reminder-based interventions in improving patient adherence to scheduled appointments.

In this study, SMS reminders were preferred over phone calls by a significant proportion of participants (204 out of 493). The impact of SMS reminders was notable, with an increase in appointment attendance rates paralleling findings from prior research [18,26]. However, the effectiveness of SMS reminders is not universal [13,27]. These variations could be attributed to factors such as patients’ perception of the appointment value, the patient-hospital relationship, standard practices in patient reminders, and demographic factors. The timing of SMS reminders emerged as a pivotal aspect of this intervention. Reminders sent either too early or too late might not effectively enhance attendance rates [18]. In our study, reminders were sent 48 hours prior to the appointment, except on weekends when they were sent 72 hours in advance. This approach aligned with several existing studies, and results suggested that reminders sent closer to the appointment date may assist in reducing forgetfulness. However, the variation in reminder timings across different studies does not conclusively impact the rate of non-attendance, indicating flexibility in timing does not sacrifice effectiveness. This study ensured that the SMS content was concise and clear, and contained essential information like appointment date, time, and location, along with a request for confirmation. The content of SMS reminders plays a critical role in their success. This approach is supported by the literature, emphasising the influence of message content on attendance rates [14]. The effectiveness of reminders could be enhanced by inducing an emotional response, such as guilt, although care must be taken to customise content to patients’ cultural and age-specific contexts.

This study also explored how various factors, such as age, gender, and marital status, influence the effectiveness of SMS reminders. Age emerged as a significant factor, with patients aged 21 to 40 years responding more favourably to SMS reminders. This age-specific preference aligned with broader research trends, suggesting younger demographics are more inclined towards text-based communication [28,29]. However, gender did not significantly influence the effectiveness of SMS reminders in our study, despite prior research indicating potential differences in appointment adherence between men and women [26,30]. This discrepancy suggested that the influence of gender on reminder effectiveness might be context-specific and not universally applicable. Moreover, a high proportion of married patients who received SMS reminders attended their appointments. This finding might reflect the additional social support married individuals often have, aiding appointment adherence.

Apart from other factors, it is important to consider the balance between frequency and content while implementing reminder strategies. While reminders are crucial, excessive messaging can lead to patient fatigue or annoyance, potentially diminishing the intervention’s effectiveness [16]. This study adhered to a policy of sending a single reminder per appointment, a strategy supported by research indicating that multiple reminders can improve attendance rates [8,30].

Further, in this study, phone call reminders were also employed as a method to enhance attendance at radiology appointments. Of the 493 participants, 289 chose phone call reminders over SMS. The effectiveness of phone calls in improving patient attendance was noteworthy, aligning with other studies that highlighted their potential in reducing appointment non-attendance. The study by Satiani et al. (2009) [23] revealed that automated phone reminders, in some cases, increased non-attendance, possibly due to higher inherent compliance in certain patient groups. This finding emphasises the nuanced nature of reminder effectiveness. The implementation process for phone call reminders involved scheduling appointments based on patient preferences, with reminders typically sent 48 hours in advance, or 72 hours for weekend appointments. The timing of reminders, a critical factor, has varied across different studies, with no definitive correlation found between timing and reduced non-attendance rates. Customised phone call reminders were predominantly used in this study. These calls, made without recording the conversation, focused on confirming appointment details with the patient. Existing research, such as that by Parikh et al. (2010) [22], has suggested that personalised reminders from clinic staff might be more effective than automated reminders, potentially due to the personal connection established. When reminder frequency is considered, this study indicated that a single phone call might not always be sufficient to influence patient attendance. While the impact of multiple reminders remains inconclusive, their potential to enhance attendance rates warrants consideration. Phone call reminders were particularly effective for patients aged 41-60 years, echoing findings from other studies that identified age as a significant factor in intervention effectiveness. This result reflects older patients’ preference for traditional communication methods like phone calls. Gender differences in response to phone call reminders were not significant, with similar attendance rates for both male and female patients after the intervention. This observation is consistent with other research, such as that of Bos et al. (2005), who also reported no significant gender difference in appointment attendance with reminder interventions [27]. Married patients had a slightly higher responsiveness to phone call reminders. This preference among married individuals, who are often middle-aged, might reflect their inclination towards traditional communication methods. The comparative analysis emphasised the influence of demographic, geographic, and clinical variables on patient attendance rates in response to the intervention. The findings provide insights into the factors that most significantly impacted patient attendance at the hospital, both before and after the implementation of the intervention reminders.

The intervention involved using SMS to send reminders to patients, but this approach had limitations. The inability to verify message receipt and whether messages were read posed challenges, as did accounting for calls to reschedule appointments. Potential bias due to the researcher's involvement in intervention development and evaluation was noted, although the researcher only guided recruitment and data collection processes. The analysis of anonymous data was further reviewed. Also, the use of convenience sampling, where patients were approached during their visit to the radiology department, may introduce selection bias, potentially underrepresenting certain groups such as those with transportation issues or those who do not visit the hospital regularly. Another limitation was that the study did not investigate the cost-effectiveness of SMS versus telephone reminders, despite SMS being cheaper. An ethical concern included in this study was the risk of SMS messages being read by others, potentially causing embarrassment to the patients. Additionally, it was unclear whether appointment attendance was influenced by SMS reminders or patients' prior decisions.

## Conclusions

This study assessed the impact of interventions on reducing missed appointments in a radiology department. The research involved reminding patients of their upcoming appointments through either a phone call or SMS, based on their preference. The descriptive analysis indicated that male adults and patients of Saudi nationality with a degree exhibited increased attendance rates following the intervention. Among the reminder methods used, phone calls emerged as the most effective in decreasing missed appointments. The analysis highlighted that attendance frequencies for patients reminded by phone calls varied between 35% and 85%, whereas those reminded via SMS showed a 15-65% attendance range. In the inferential analysis, marital status and distance from the hospital were identified as significant variables associated with attendance. The chi-square analysis comparing pre- and post-intervention attendance rates revealed significant associations in certain categories. Attendance rates of female patients, those with at least secondary education, and those with single or divorced relationship status exhibited significant shifts. Further, the analysis suggested that patients referred from other hospitals and those living more than 35 km from the hospital were more likely to miss their appointments than other patients regardless of the intervention. Patients who were not required to pay for services demonstrated higher attendance rates. The chi-square analysis elucidated variables that significantly correlated with attendance rates before and after the intervention. These findings emphasise the effectiveness of phone call reminders in reducing patient non-attendance and highlight the influence of demographic, clinical, and other factors on attendance rates. The results also support the notion that personalised scheduling coupled with reminder systems can effectively encourage patients to attend their appointments. These results offer valuable guidance for developing more effective strategies to improve appointment attendance in hospital settings.
